# Effects of different doses of intranasal dexmedetomidine on related complications and parents’ satisfaction in anesthetized children: a systematic review

**DOI:** 10.1186/s12887-024-04832-w

**Published:** 2024-05-31

**Authors:** Wei Hu, Ming Wang, Fei Sun

**Affiliations:** Department of Anesthesiology, Children’s Hospital of Nanjing Medical University, No. 72 Guangzhou Road, Nanjing, 210008 Jiangsu Province China

**Keywords:** Dexmedetomidine, Delirium, Agitation, Children, Randomized controlled trial, Anesthesia

## Abstract

**Background:**

Agitation/delirium is commonly seen in children after anesthesia, and a proper dose of dexmedetomidine can prevent this complication. This study aimed to investigate the effects of different doses of Dexmedetomidine (DEX) on agitation/delirium and other complications in anesthetized children, providing clinical evidence for dose recommendations of DEX.

**Methods:**

This study was conducted based on the Preferred Reporting Items for Systematic Reviews and Meta-Analyses (PRISMA). A systematic search was conducted in the Cochrane Library, PubMed, Web of Science, and EMBASE. Two independent researchers performed literature screening, data extraction, and assessed the methodological quality. Data analysis was conducted using R and STATA 16.0.

**Results:**

In the final analysis, 20 randomized controlled trials (RCTs) involving 2521 children were included. The results showed that in comparison to normal saline, 1 µg/kg, 1.5 µg/kg, and 2 µg/kg intranasal DEX significantly reduced the incidence of post-anesthetic emergence agitation in children with the most effective dose being 2 µg/kg (SUCRA = 0.91). Compared with normal saline, 1 µg/kg, 1.5 µg/kg, and 2 µg/kg intranasal DEX reduced patient’s need for postoperative analgesia, with the most effective dose being 1.5 µg/kg (SUCRA = 0.78). However, 1 µg/kg DEX performed the best in reducing Pediatric Anaesthesia Emergence Delirium (PAED) Scale score (SUCRA = 0.88).

**Conclusion:**

Compared with normal saline, intranasal administration of 2 µg/kg DEX and 1.5 µg/kg DEX are the optimal doses to reduce the incidence of agitation and the need for postoperative pain relief in children under general anesthesia. Given effectiveness and safety, intranasal use of 1 µg/kg DEX appears to be the most effective dosage for anesthetized children.

**Supplementary Information:**

The online version contains supplementary material available at 10.1186/s12887-024-04832-w.

## Background

It is common for pediatric patients to feel nervous and anxious during anesthesia and operation, with the incidence of severe preoperative anxiety reaching up to 60% [1]. This may be due to the unfamiliar operating room environment and the fear of being separated from their parents before operation. In this case, the pediatric patients tend to experience emotional outbursts and refuse to cooperate, which not only increases the incidence of anesthesia risks and adverse events, but also increases the negative emotions of the patients and their family members [2]. Research indicates that preoperative anxiety can result in postoperative agitation in children, as well as have adverse psychological and behavioral effects [3]. Emergence agitation (EA) is a postoperative complication that occurs during the emergence period of general anesthesia. It refers to a state in which pediatric patients experience dissociation between their consciousness and behavior. EA can trigger severe complications such as vomiting, aspiration, and even laryngospasm [4]. Emergence delirium (ED) represents a behavioral disorder in children characterized by crying, fear, instability, and disorientation in the early stages of anesthesia recovery. Improper anesthesia techniques and postoperative airway obstruction and pain significantly contribute to the occurrence of these complications [5, 6]. Therefore, the use of effective interventions to promote cooperation, ensure clinical safety, enhance overall comfort, and reduce postoperative adverse reactions has become a focal point in anesthesia research and efforts.

Currently, numerous interventions have been studied for managing EA and emergence delirium, including pharmacological and behavioral approaches [7]. Among these interventions, Dexmedetomidine (DEX)is a highly selective α2-adrenergic receptor agonist. It possesses sedative, hypnotic, anxiolytic, and analgesic properties while maintaining a short half-life, providing easily reversible sedation without respiratory depression [8]. It achieves maximum average blood drug concentration within approximately 37 min after administration via the intranasal route, which is well-tolerated without causing pain or irritation. In 1999, it was first approved by the FDA for sedation in critically ill patients. DEX provides sedation and anxiolysis by acting on α2 receptors located in the pontine locus coeruleus, and it exerts dose-dependent analgesic effects through binding to α2 receptors in the dorsal horn and upper spinal cord. In recent years, DEX has been widely used for preventing EA and ED in pediatric patients, although dose-related side effects such as hypotension and bradycardia may increase [9].

DEX holds significant potential in various clinical scenarios. To ensure the safe use of DEX, it is necessary to carefully determine appropriate dosages. Therefore, this systematic review aimed to observe the effects of intranasal DEX at different doses on the occurrence of emergence agitation or delirium in children undergoing general anesthesia.

## Methods

This study was conducted according to the Preferred Reporting Items for Systematic Reviews and Meta-Analyses (PRISMA) [10], and this study was registered in the International Prospective Register of Systematic Reviews (PROSPERO) database (https://www.crd.york.ac.uk/PROSPERO), with the ID of CRD42023441872.

### Inclusion and exclusion criteria

Based on the definitions of participants, interventions, comparators, outcomes, and study design (PICOS) in the Cochrane Handbook for Systematic Reviews of Interventions, the following inclusion and exclusion criteria were formulated.

#### Inclusion criteria

(1) Participants (P): Children assessed as American Society of Anesthesiologists (ASA) physical status I-III, < 12 years old, scheduled for examination or surgery under general anesthesia, and without known allergies to DEX.

(2) Intervention (I): Experimental group receiving intranasal administration of DEX.

(3) Comparator (C): Control group receiving different doses of intranasal DEX or normal saline compared to the experimental group.

(4) Outcomes (O): Incidence of emergence delirium, Pediatric Anesthesia Emergence Delirium (PAED) score, Postanesthesia care unit (PACU) stay time, postoperative length of hospital stay, postoperative gastrointestinal adverse reactions, parent’s satisfaction, analgesics, and postoperative pain scores.

(5) Study Types (S): Randomized controlled trials.

#### Exclusion criteria

(1) DEX combined with other narcotics.

(2) Reviews, expert consensus, in vitro studies, animal experiments, case reports, letters, and replies.

(3) Data that contain significant errors or missing information, and it is not possible to contact the corresponding author of the literature.

### Search strategy

We conducted a systematic search in PubMed, Embase, Cochrane Library, and Web of Science databases for relevant studies published from the inception of databases up to June 25, 2023. The keywords used were (“Dexmedetomidine”) and (“Child” or “Adolescent” or “Pediatrics” or “Youth” or “Teen”), without restrictions on region or language. Table S1 describes the detailed search strategies for each database.

### Literature screening and data extraction

Two researchers (Hu Wei and Wang Ming) strictly followed the inclusion criteria to screen articles and extract data and cross-checked their findings. In cases of disagreement, a third researcher (Sun Fei) joined the discussion for consensus.

The retrieved articles were imported into Endnote X9 for management and screened by two researchers (Hu Wei and Wang Ming). Duplicate articles were firstly removed manually and by machine marking. Then, the two researchers screened the remaining articles independently by reading the title, abstracts and full texts according to the pre-designed inclusion and exclusion criteria. After obtaining the full texts of the articles meeting the inclusion criteria, two researchers independently extracted the following data: (1) basic study characteristics such as authors, publication year, country, patient source, age, gender, and sample size; (2) key elements for assessing bias risk; and (3) outcome measures.

### Risk of bias in included studies

The quality assessment of included studies was independently conducted by two researchers (Hu Wei and Wang Ming) using Version 2 of the Cochrane tool (RoB2) [11]. Any discrepancies were resolved through consultation with a third reviewer (Sun Fei). The assessment comprised five domains: bias in the randomization process, bias due to deviations from intended interventions, bias caused by missing outcome data, bias in outcome measurement, and bias in the selection of reported results. Each included study was evaluated according to the above criteria. If the five domains of a study were assessed as low risk of bias (RoB), the overall RoB was evaluated as low risk. If all five domains were not assessed as high RoB, but one of the domains was rated as having a possibility of RoB, the overall RoB was classified as unclear risk. If one of the five domains was assessed as high RoB, or multiple domains were graded as unclear RoBs and have a great impact on the credibility of the study results, the overall RoB was high [12].

### Statistical analysis

All the data analyses in this study were performed by Stata 15.1 and R software (VER. 4.0.3) and Rstudio. For dichotomous variables, we calculated the risk ratio (RR) with corresponding 95% confidence intervals (CI). For continuous variables with the same scale and unit, we used the mean difference (MD) with corresponding 95%CI. In cases where the units were not uniform or different scales were used, we employed the standardized mean difference (SMD) with corresponding 95%CI. Given the heterogeneity among different experiments, a Bayesian random-effects model was employed for multiple comparisons of various treatment regimens of intranasal DEX on postoperative agitation or delirium and other complications in pediatric patients under general anesthesia. We employed a Markov Chain Monte Carlo method for modeling, with four Markov chains running simultaneously and setting the number of annealing iterations to 20,000. The modeling process was completed after 50,000 simulation iterations. The deviance information criterion (DIC) was used to compare whether the overall consistency and inconsistency of model fitting are unified. If the DIC value is less than 5, the overall consistency and inconsistency are unified, and vice versa. If there were closed loops, we further analyzed local consistency using node-splitting. Additionally, we ranked the interventions based on Surface Under the Cumulative Ranking curve (SUCRA), which shows the likelihood of each treatment being the best invention and generated a league table for comparison of treatment efficacy among various interventions [13, 14]. The closer the SUCRA value is to 100%, the higher the ranking of a therapy, and the better the intervention effect. The funnel plot was used to visualize the publication bias of the study.

## Results

### Systemic Retrieval results

**Fig. 1:**
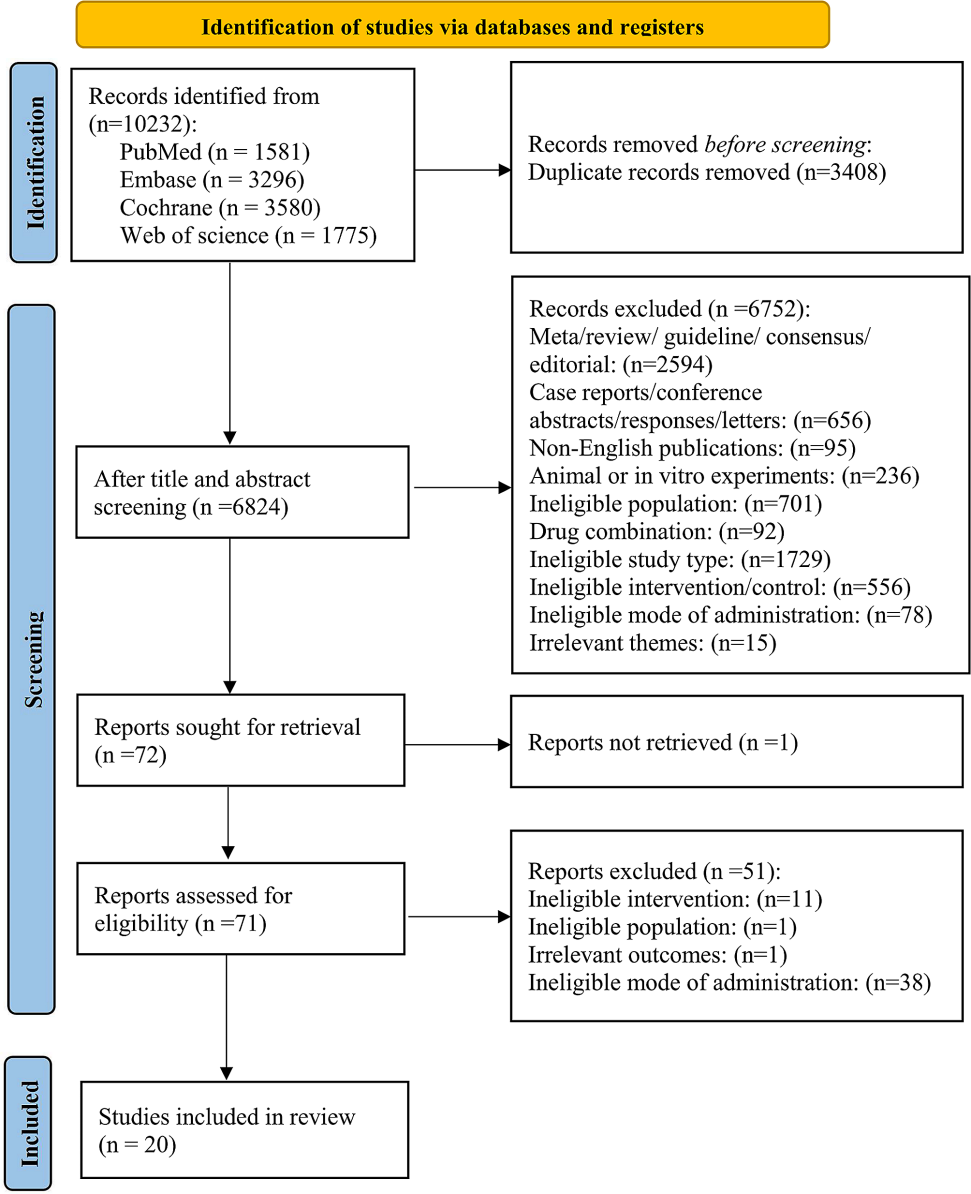
Flowchart of literature screening process

A total of 10,232 articles were obtained from the databases. After removing duplicates (*n* = 3408), 6824 studies remained. By excluding irrelevant articles (*n* = 6752) based on titles and abstracts, 72 articles were retained. Among these, full text could not be found for one article, and after reading the remaining 71 articles, 51 articles that did not meet the inclusion criteria were excluded. Finally, 20 RCTs were included in this network meta-analysis [15–34]. The flowchart is shown in Fig. 1.

### Basic Information of included studies

**Fig. 2:**
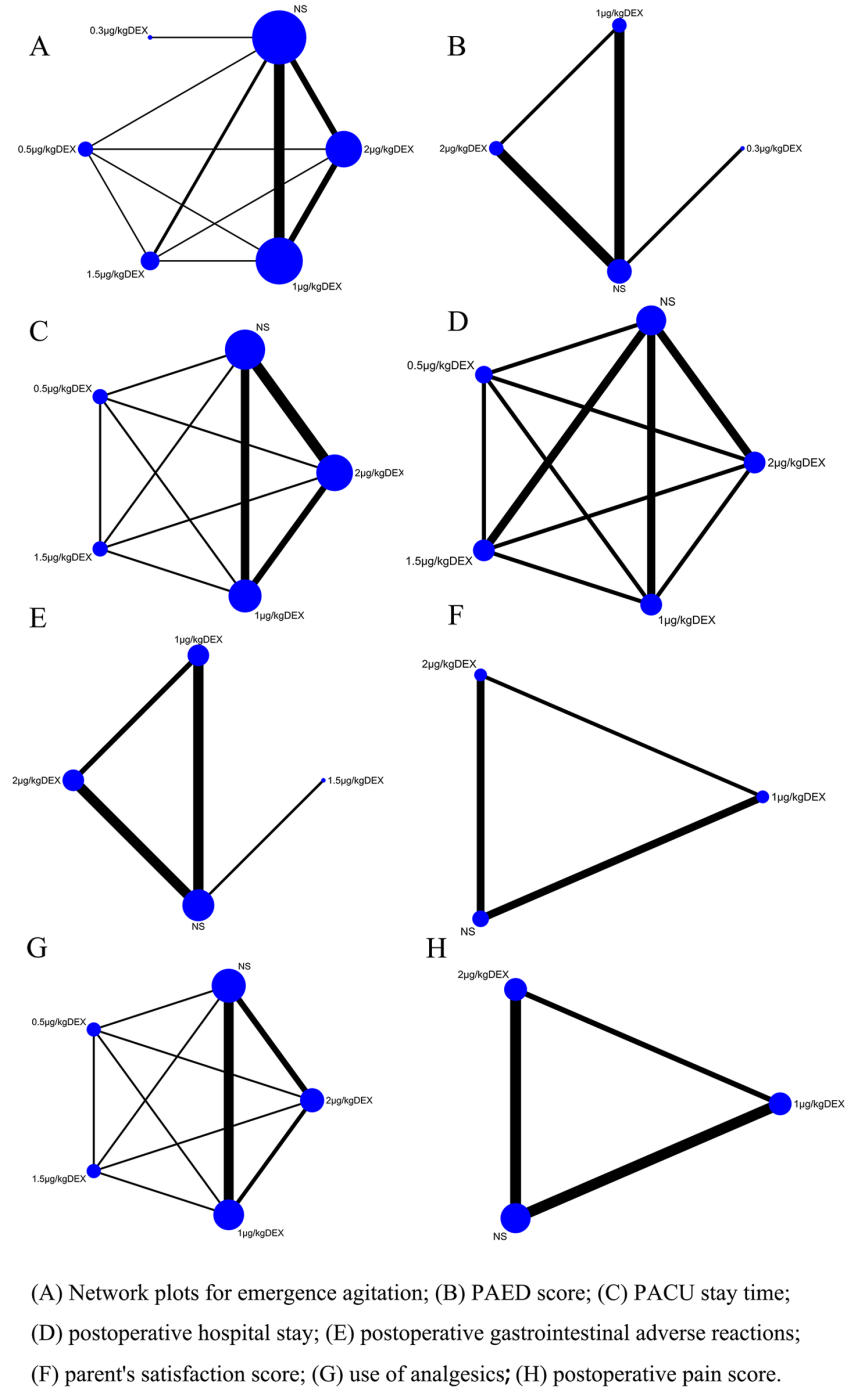
Network plots. (**A**) Emergence agitation; (**B**) PAED score; (**C**) PACU stay time; (**D**) Postoperative length of hospital stay; (**E**) Postoperative gastrointestinal adverse reactions; (**F**) Parental satisfaction; (**G**) Analgesics (**H**) Postoperative pain scores

A total of 2521 children younger than 18 years old from 20 RCTs were included in this network meta-analysis, with a sex ratio (Man/Female) of 1607: 914. Among the included studies, 10 articles were from China [20, 24–26, 28, 30–34], 4 from India [19, 22, 23, 29], 3 from Egypt [17, 18, 21], 1 from United States of America [27], 1 from Australia [15] and 1 from Saudi Arabia [16]. Table 1 presents the baseline characteristics of each trial, and Fig. 2 displays the network plot of different interventions on the outcomes.

**Table 1:** Baseline characteristics of included literature

Author	Year	Country	Case source	Study type	Intervention measures	Control measures	Sample size	Age	Male/female
Pestieau et al.	2011	USA	Single-center	Double-blind RCT	1 µg/kg DEX 2 µg/kg DEX	Normal saline	101	6 m-6y	66/35
Gyanesh et al.	2013	India	Single-center	Double-blind RCT	1 µg/kg DEX	Normal saline	150	1–10	98/52
Wang et al.	2013	China	Single-center	RCT	1 µg/kg DEX	2 µg/kg DEX	40	3–6	26/14
Yao et al.	2014	China	Single-center	Double-blind RCT	1 µg/kg DEX 2 µg/kg DEX	Normal saline	90	3–7	54/36
Abdelaziz et al.	2016	Saudi Arabia	Single-center	Double-blind RCT	1 µg/kg DEX	Normal saline	98	1–7	52/46
Ali et al.	2016	Egypt	Single-center	Double-blind RCT	0.3 µg/kg DEX	Normal saline	90	3–6	56/34
Lin et al.	2016	China	Single-center	Single-blind RCT	1 µg/kg DEX 2 µg/kg DEX	Normal saline	90	2–7	48/42
Sidhu et al.	2016	India	Single-center	Double-blind RCT	2 µg/kg DEX	Normal saline	105	2–9	83/22
Abd El-Hamid et al.	2017	Egypt	Single-center	Double-blind RCT	1 µg/kg DEX	Normal saline	86	3–7	44/42
Li et al.	2018	China	Single-center	Double-blind RCT	1 µg/kg DEX 2 µg/kg DEX	Normal saline	90	2–7	50/40
Anupriya et al.	2019	India	Single-center	Double-blind RCT	2 µg/kg DEX	3 µg/kg DEX	59	1–8	48/11
Bi et al.	2019	China	Single-center	Double-blind RCT	1 µg/kg DEX	Normal saline	40	6–48 m	29/11
Yao et al.	2020	China	Single-center	Double-blind RCT	2 µg/kg DEX	Normal saline	153	2–6	100/53
Zhang et al.	2020	China	Single-center	RCT	1.5 µg/kg DEX	Normal saline	134	1-4y	57/77
Gupta et al.	2021	India	Single-center	RCT	1 µg/kg DEX	Normal saline	105	2–8	54/51
Lee-Archer et al.	2021	Australia	Single-center	RCT	1 µg/kg DEX 2 µg/kg DEX	Normal saline	247	2–7	152/95
Ali et al.	2022	Egypt	Single-center	Double-blind RCT	3 µg/kg DEX	4 µg/kg DEX	50	1–8	19/31
Lei et al.	2022	China	Single-center	Double-blind RCT	0.5 µg/kg DEX 1 µg/kg DEX 1.5 µg/kg DEX 2 µg/kg DEX	Normal saline	300	1–10	278/22
Shen et al.	2022	China	Single-center	Double-blind RCT	2 µg/kg DEX	Normal saline	373	0–12	221/142
Yao et al.	2022	China	Single-center	Double-blind RCT	1 µg/kg DEX	Normal saline	120	2–6	72/48

### Risk of bias assessment results

All the 20 trials described the process of sequence generation. No reports were considered to have a high or unclear RoB in randomization process, deviations from intended interventions or missing outcome data. In the measurement of the outcome, 6 studies [16, 21, 22, 29, 32, 33] (28.6%) had an unclear RoB. Regarding the selective reporting, 8 studies [21–24, 26, 27, 31, 33] (47.6%) had a moderate RoB.

In terms of the overall bias, 42.9%, 57.1%, and 0% of studies had a low RoB, moderate RoB, and high RoB, respectively. The risks were mainly caused by the measurement methods of the outcome in 6 articles [16, 21, 22, 29, 32, 33] and selective reporting in 8 articles [21–24, 26, 27, 31, 33].

**Fig. 3:**
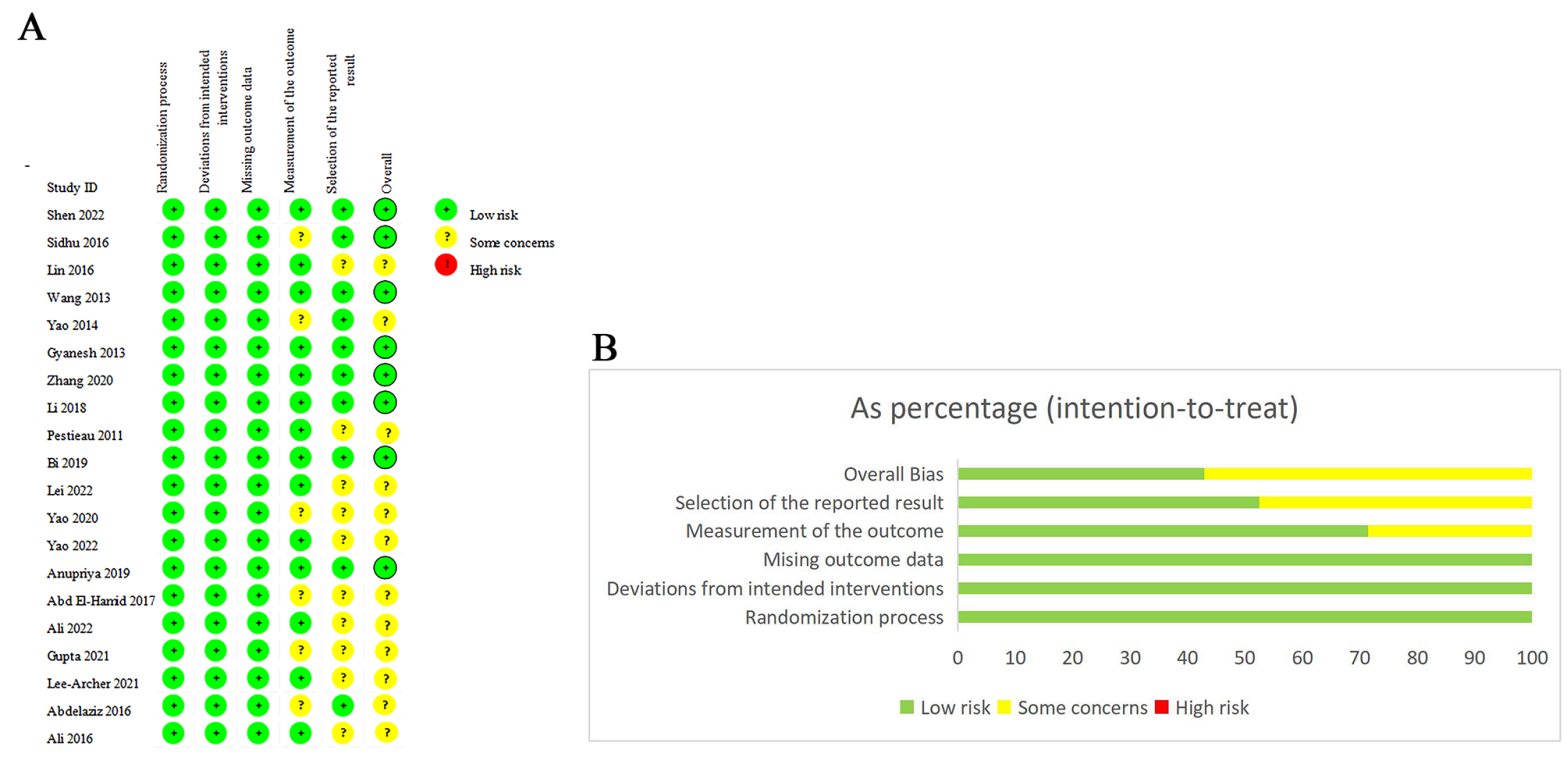
Risk of bias assessment

According to the quality assessment tool, all studies were of good quality. The RoB of the included studies is shown in Fig. 3.

### Network Meta-analysis

The network plot is shown in Fig. 2: emergence agitation (A), PAED score (B), PACU stay time (C), postoperative length of hospital stay (D), postoperative gastrointestinal adverse reactions (E), parent’s satisfaction score (F), use of analgesics (G) and postoperative pain score (H).

Each dot represents different interventions, and the size of the dot represents the number of articles involved in the relevant intervention. The larger the dot, the more studies involved in the relevant intervention. The line indicates the comparison between interventions, and the thickness of the line indicates the number of related articles. The thicker the line, the more articles involved in the two related interventions.

#### Emergence agitation

Nine studies [16, 18, 20, 21, 24, 26, 27, 32, 34] reported the incidence of emergence agitation. The network meta-analysis revealed that after compared to normal saline, intranasal DEX intervention at 1 µg/kg [RR = 0.305, 95%CI= (0.154, 0.579)], 1.5 µg/kg [RR = 0.276, 95%CI= (0.085, 0.905)], and 2 µg/kg [RR = 0.163, 95%CI= (0.059, 0.373)] significantly decreased the incidence of emergence agitation. However, 0.5 µg/kg of DEX had a higher incidence of emergence agitation compared to 2 µg/kg [RR = 4.49, 95%CI= (1.124, 24.37)]. There was no significant difference between other pairwise interventions (Table S2).

#### PAEDs

Six studies reported the PAEDs [16, 18, 25, 28, 31, 33]. The meta-analysis revealed that compared to normal saline, intranasal DEX at 1 µg/kg [MD=-3.20, 95%CI= (-5.39, -1.00)] significantly reduced the PAEDs, and the difference was statistically significant. However, there was no statistically significant difference between DEX at 2 µg/kg [MD=-1.83, 95%CI= (-3.92,0.26)] or 0.3 µg/kg [MD=-0.14, 95%CI= (-3.80,3.52)] and normal saline. No statistically significant differences were observed in the pairwise comparison of different doses (Table S3).

#### PACU stay time

Six studies [16, 24, 26, 28, 32, 33] reported the PACU stay time. The meta-analysis revealed that compared to normal saline, intranasal DEX at 1.5 µg/kg [SMD = 0.83, 95%CI= (0.17, 1.5)] and 2 µg/kg [SMD = 0.59, 95%CI= (0.15, 1.02)] significantly prolonged the PACU stay time. Pairwise comparisons were conducted between different doses of DEX, and only the differences between intranasal DEX at 0.5 µg/kg and DEX at 1.5 µg/kg [SMD=-1.03, 95%CI= (-1.78, -0.28)] and between DEX at 0.5 µg/kg and 2 µg/kg [SMD=-0.79, 95%CI= (-1.46, -0.12)] were statistically significant. However, there was no statistically significant difference between DEX at 0.5 µg/kg [SMD=-0.2, 95%CI= (-0.86, 0.46)] or 1 µg/kg [SMD = 0.19, 95%CI= (-0.27, 0.65)] and normal saline (Table S4).

#### Postoperative length of hospital stay

Four studies [21, 24, 28, 34] reported postoperative length of hospital stay. The meta-analysis revealed no statistically significant differences (Table S5).

#### Postoperative gastrointestinal adverse reactions

Seven studies [16, 21, 25, 26, 28, 33, 34] reported the incidence of postoperative gastrointestinal adverse reactions. Among them, five studies [16, 25, 26, 28, 34] reported vomiting and three studies [21, 33, 34] reported vomiting and nausea. The meta-analysis revealed no statistically significant differences (Table S6).

#### Parental satisfaction

Three studies [31–33] reported parent’s satisfaction. The overall meta-analysis demonstrated that at doses of 1 µg/kg [SMD = 1.46, 95%CI= (0.57, 2.36)] and 2 µg/kg [SMD = 2.21, 95%CI= (1.3, 3.1)], intranasal DEX showed an increase in parent’s satisfaction scores compared to normal saline. No statistically significant differences were observed in the pairwise comparison of different doses (Table S7).

#### Analgesics

Six studies [16, 20, 21, 24, 27, 28] reported the use of analgesics, of which two studies [14, 25] focused on the use of acetaminophen, one study [20] on the use of remifentanil, one study [21] on 1% lidocaine, one [24] on nalbuphine, and one [28] on fentanyl. The meta-analysis demonstrated that at doses of 1 µg/kg [RR = 0.22, 95%CI= (0.082, 0.569)], 1.5 µg/kg [RR = 0.128, 95%CI= (0.011, 0.978)], and 2 µg/kg [RR = 0.241, 95%CI= (0.061, 0.656)], intranasal DEX improved the use of analgesics compared to normal saline. However, no statistically significant difference was found between 0.5 µg/kg DEX [RR = 0.283, 95%CI= (0.042, 1.791)] and normal saline. No statistically significant differences were observed in the pairwise comparison of four doses (Table S8).

#### Postoperative pain scores

Six studies [16, 21, 25, 26, 28, 33] reported on postoperative pain. Two of these studies [14, 19] used the Face, Legs, Activity, Cry and Consolability (FLACC) scale to evaluate pain, three [25, 26, 33] adopted the Children’s Hospital of Eastern Ontario Pain Scale (CHEOPS), and one [28] employed the Wong-Baker Faces Pain Rating Scale. No statistically significant differences were found (Table S9).

### SUCRA Ranking results

**Fig. 4:**
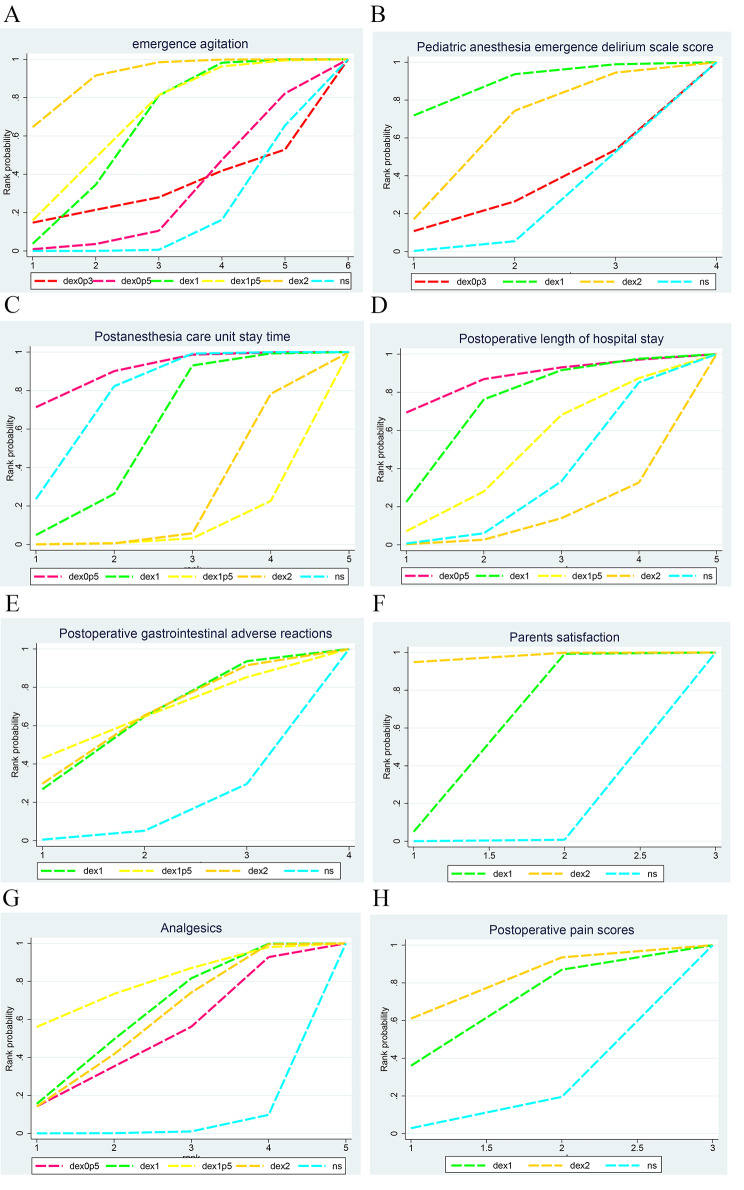
Ranking plots. (**A**) Emergence agitation; (**B**) PAED score; (**C**) PACU stay time; (**D**) Postoperative length of hospital stay; (**E**) Postoperative gastrointestinal adverse reactions; (**F**) Parental satisfaction; (**G**) Analgesics (**H**) Postoperative pain scores

The ranking results of the SUCRA showed that the top three regimens for emergence agitation were 2 µg/kg DEX, 1.5 µg/kg DEX, and 1 µg/kg DEX, then 0.3 µg/kg DEX, 0.5 µg/kg DEX, and NS successively; the top three regimens for PAEDs were 1 µg/kg DEX, 2 µg/kg DEX, and 0.3 µg/kg DEX, with NS ranking the last; the top three regimens for PACU stay time were 0.5 µg/kg DEX, NS, and 1 µg/kg DEX, then 2 µg/kg DEX, and 1.5 µg/kg DEX successively; the top three regimens for postoperative hospital stay were 0.5 µg/kg DEX, 1 µg/kg DEX, and 1.5 µg/kg DEX, then NS, and 2 µg/kg DEX; The top three regimens for reducing postoperative gastrointestinal adverse reactions were 1.5 µg/kg DEX, 2 µg/kg DEX, and 1 µg/kg DEX, with NS ranking the last; the top three regimens for parental satisfaction were 2 µg/kg DEX, 1 µg/kg DEX, and NS; the top three regimens for requiring analgesics were 1.5 µg/kg DEX, 1 µg/kg DEX and 2 µg/kg DEX, then 0.5 µg/kg DEX, and NS; the top three in the postoperative pain score were 2 µg/kg DEX, 1 µg/kg DEX, and NS. Details are shown in Fig. 4 and Table S10. The cumulative probability suggests that there may be shorter PACU stay and shorter postoperative hospital stay with 0.5 µg/kg DEX; lower PAED scores with 1 µg/kg DEX; fewer analgesics and lower incidence of postoperative gastrointestinal adverse reactions with 1.5 µg/kg DEX; less emergence agitation, higher parent’s satisfaction, and less postoperative pain with 2 µg/kg DEX.

### Publication bias and inconsistency

To assess publication bias, we created funnel plots using STATA software for outcome measures that were included in at least five studies. Different colors were used to indicate the comparisons between interventions. The funnel plot showed that there was no potential publication bias in the outcomes. The Deviance Information Criterion (DIC) and node-splitting analysis indicated no statistical inconsistency. Details are provided in Figure S1.

## Discussions

To the best of our knowledge, this is the first Bayesian network meta-analysis focusing on the effects of different doses of DEX on postoperative agitation or delirium and other outcomes in anesthetized pediatric patients. In this systematic review and network meta-analysis, we incorporated direct and indirect evidence from 20 randomized controlled trials to compare the effects of intranasal DEX at doses of 0.3 µg/kg, 0.5 µg/kg, 1 µg/kg, 1.5 µg/kg, and 2 µg/kg on postoperative agitation or delirium and other outcomes in pediatric patients undergoing anesthesia. Our results indicate that the application of DEX in pediatric anesthesia is effective in reducing emergence agitation, PAED scores, PACU stay time and the use of additional anesthetics, and improving parent’s satisfaction, compared with the control group. In the comparison between different doses, we found that 0.5 µg/kg can significantly shorten the PACU stay time and lower the incidence of emergence agitation. The cumulative probability results show that in comparison of different DEX doses, 0.5 µg/kg DEX is associated with the shortest PACU stay, 1 µg/kg DEX with the lowest PAED scores; 1.5 µg/kg DEX with the least use of analgesics, and 2 µg/kg DEX with the lowest incidence of emergence agitation.

Zhang et al.‘s research revealed that nasal administration of DEX can reduce the incidence of agitation after inhalation anesthesia in pediatric patients, but the optimal dose is still uncertain [35]. Our research has shown that compared with the control group, 1 µg/kg DEX, 1.5 µg/kg DEX and 2 µg/kg DEX can significantly reduce the incidence of emergence agitation, with 2 µg/kg DEX showing the best performance. Aerosolized intranasal dexmedetomidine is an anesthetic strategy that can be used for sedation in pediatric patients without the need for vascular access during diagnostic procedures. Miller’s study showed that increasing the dose from 1 µg/kg to 2 µg/kg halved the time to mean arterial plasma concentration and almost doubled the mean peak plasma concentration. Higher doses of DEX may provide better sedation [36]. Besides, compared with 2 µg/kg DEX, 0.5 µg/kg DEX increased the incidence of agitation. Therefore, to reduce the incidence of emergence agitation in clinical practice, relatively higher doses of DEX are required under safe conditions.

We also found that 1 µg/kg DEX significantly reduced PAED scores, which may contribute to its neuroprotective properties as shown in a previous study [37]. DEX is an α2-adrenoceptor agonist [38], which exerts neuroprotective effects by α2-adrenoceptor-dependent or independent modulation of neuroinflammation, apoptosis, oxidative stress, and synaptic plasticity [37].

A meta-analysis by Zhang et al. demonstrated that DEX can reduce the use of postoperative analgesics in adult patients undergoing general anesthesia [39]. Similarly, we observed that all three doses of DEX (1 µg/kg, 1.5 µg/kg, and 2 µg/kg) reduced the use of analgesics in the pediatric population compared with the control group, Furthermore, we found that both 1 µg/kg and 2 µg/kg DEX exhibited similarly favorable results in terms of parent’s satisfaction. The cumulative probability results suggest that 1.5 µg/kg DEX may be the most ideal for reducing analgesic use, while 2 µg/kg DEX may provide the highest level of parent’s satisfaction. Infants and children experience pain in a manner similar to adults, with emotional factors particularly increasing their perception of pain, which is exactly where sedatives play a role [40, 41]. Although we couldn’t exactly explain the dose differentiation in pain due to the inclusion of patients of different age or disease background, and further research is needed to determine the effects of different doses on patients’ pain, it cannot be denied that 1 µg/kg, 1.5 µg/kg and 2 µg/kg DEX have an effect on pain relief in pediatric patients.

We also found that both 1.5 µg/kg and 2 µg/kg DEX prolonged PACU stay compared with normal saline. In addition, when comparing different doses, both 1.5 µg/kg and 2 µg/kg significantly prolonged PACU stay compared with 0.5 µg/kg. In contrast to our results, the study by Xu et al. showed that DEX did not reduce PACU stay compared with the control group [42]. This may be due to the relatively short duration of intranasal dexmedetomidine administration and outpatient surgery in some of the included studies.

The most common adverse events induced by DEX are hypotension and bradycardia, and cardiac arrest may occur when high-dose DEX is used [43]. Li et al. reported that 3 female patients suffered from bradycardia during intravenous (IV) dexmedetomidine injection under general anesthesia [44]. The use of dexmedetomidine can reduce the ventilatory responses to hypoxia and hypercapnia, and interact with the peripheral and central control of respiration, resulting in respiratory inhibition [45]. Although our results showed no significant difference in the incidence of postoperative gastrointestinal adverse reactions, the length of hospital stay, and postoperative pain score between the DEX group and the control group, the potential side effects of DEX should not be neglected.

This study has some limitations that currently cannot be resolved. Although we have carried out a comprehensive and systematic search, the relevant literature retrieved is still limited, so the age of the included population, the source of the drug, types of surgery and anesthesia, and the different administration times of DEX were not fully discussed. In addition, given the limitation of quantity, most of the treatment modalities included in this study were not directly compared, thus indirect comparisons played a crucial role in our study. Furthermore, although we carefully included the retrieved studies according to randomized controlled trials in the admission stage, the RCTs eventually included were small samples, single-center studies, which may lead to overestimating the efficacy and underestimating the risk of adverse reactions. In addition, there were few doses involved in the included intervention, so it was unfeasible to conduct multiple comparisons, and the effect of anesthesia was mostly affected by the patient’s own physical condition, so the results should be interpreted with caution.

## Conclusions

In summary, our results have proved that intranasal DEX exhibits satisfactory sedative and analgesic effects, which can improve the quality of postoperative recovery in children under general anesthesia without increasing gastrointestinal adverse reactions. Therefore, given drug effectiveness and safety, a dose of 1 µg/kg intranasal DEX appears to be most effective for anesthetized children. More large-sample, multicenter clinical studies are warranted to further supplement the conclusions of this study.

### Electronic supplementary material

Below is the link to the electronic supplementary material.

Supplementary Material 1

## Data Availability

The datasets used and/or analysed during the current study available from the corresponding author on reasonable request.

## Abbreviations

ASA: American Society of Anesthesiologists
CI: Confidence intervals
DIC: Deviance information criterion
DEX: Dexmedetomidine
EA: Emergence agitation
ED: Emergence delirium
MD: Mean difference
PAED: Pediatric Anesthesia Emergence Delirium
PAEDs: Pediatric anesthesia emergence delirium scale score
PACU: Postanesthesia care unit
PRISMA: Preferred Reporting Items for Systematic Reviews and Meta-Analyses
PROSPERO: Prospective Register of Systematic Reviews
RoB: Risk of bias
SMD: Standardized mean difference
SUCRA: Surface Under the Cumulative Ranking curve
RoB2: Version 2 of the Cochrane tool for assessing risk of bias in randomized trials
