# Supplementary material for: Effects of different doses of intranasal dexmedetomidine on related complications and parents’ satisfaction in anesthetized children: a systematic review
Source: BMC Pediatr. 2024 May 31;24:377. doi: 10.1186/s12887-024-04832-w (PMC11140930; doi:10.1186/s12887-024-04832-w)
Supplement: Supplementary file 1 — Supplementary Material 1 [file 12887_2024_4832_MOESM1_ESM.docx]

**Table S1:** Literature search strategy

**1.Pubmed**

| Search number | Query |
| --- | --- |
| #1 | """Dexmedetomidine""[Mesh]" Most Recent """Dexmedetomidine""[MeSH Terms]" " |
| #2 | (((((((((((((((((((Dexmedetomidine[Title/Abstract]) OR (MPV-1440[Title/Abstract])) OR (MPV 1440[Title/Abstract])) OR (MPV1440[Title/Abstract])) OR (Precedex[Title/Abstract])) OR (bxcl 501[Title/Abstract])) OR (bxcl501[Title/Abstract])) OR (cepedex[Title/Abstract])) OR (da 9501[Title/Abstract])) OR (da9501[Title/Abstract])) OR (delos[Title/Abstract])) OR (dexamedetomidine[Title/Abstract])) OR (dexdomitor[Title/Abstract])) OR (dexdor[Title/Abstract])) OR (igalmi[Title/Abstract])) OR (primadex[Title/Abstract])) OR (sedadex[Title/Abstract])) OR (sileo[Title/Abstract])) OR (tpu 006[Title/Abstract])) OR (tpu006[Title/Abstract]) """Dexmedetomidine""[Title/Abstract] OR ""MPV-1440""[Title/Abstract] OR ""MPV-1440""[Title/Abstract] OR ""MPV1440""[Title/Abstract] OR ""Precedex""[Title/Abstract] OR (""bxcl""[All Fields] AND ""501""[Title/Abstract]) OR ""bxcl501""[Title/Abstract] OR (""da""[All Fields] AND ""9501""[Title/Abstract]) OR ""delos""[Title/Abstract] OR ""dexamedetomidine""[Title/Abstract] OR ""dexdomitor""[Title/Abstract] OR ""dexdor""[Title/Abstract] OR ""igalmi""[Title/Abstract] OR ""sileo""[Title/Abstract] OR ""tpu 006""[Title/Abstract]" " |
| #3 | "(""Dexmedetomidine""[Mesh]) OR ((((((((((((((((((((Dexmedetomidine[Title/Abstract]) OR (MPV-1440[Title/Abstract])) OR (MPV 1440[Title/Abstract])) OR (MPV1440[Title/Abstract])) OR (Precedex[Title/Abstract])) OR (bxcl 501[Title/Abstract])) OR (bxcl501[Title/Abstract])) OR (cepedex[Title/Abstract])) OR (da 9501[Title/Abstract])) OR (da9501[Title/Abstract])) OR (delos[Title/Abstract])) OR (dexamedetomidine[Title/Abstract])) OR (dexdomitor[Title/Abstract])) OR (dexdor[Title/Abstract])) OR (igalmi[Title/Abstract])) OR (primadex[Title/Abstract])) OR (sedadex[Title/Abstract])) OR (sileo[Title/Abstract])) OR (tpu 006[Title/Abstract])) OR (tpu006[Title/Abstract]))" """Dexmedetomidine""[MeSH Terms] OR (""Dexmedetomidine""[Title/Abstract] OR ""MPV-1440""[Title/Abstract] OR ""MPV-1440""[Title/Abstract] OR ""MPV1440""[Title/Abstract] OR ""Precedex""[Title/Abstract] OR (""bxcl""[All Fields] AND ""501""[Title/Abstract]) OR ""bxcl501""[Title/Abstract] OR (""da""[All Fields] AND ""9501""[Title/Abstract]) OR ""delos""[Title/Abstract] OR ""dexamedetomidine""[Title/Abstract] OR ""dexdomitor""[Title/Abstract] OR ""dexdor""[Title/Abstract] OR ""igalmi""[Title/Abstract] OR ""sileo""[Title/Abstract] OR ""tpu 006""[Title/Abstract])" " |
| #4 | """Child""[Mesh]" Most Recent """Child""[MeSH Terms]" " |
| #5 | ((((((((((Child[Title/Abstract]) OR (Adolescent[Title/Abstract])) OR (Pediatrics[Title/Abstract])) OR (Children[Title/Abstract])) OR (Adolescents[Title/Abstract])) OR (Adolescence[Title/Abstract])) OR (Teen*[Title/Abstract])) OR (Youth*[Title/Abstract])) OR (Paediatric*[Title/Abstract])) OR (pediatric[Title/Abstract])) OR (pediatry[Title/Abstract]) """Child""[Title/Abstract] OR ""Adolescent""[Title/Abstract] OR ""Pediatrics""[Title/Abstract] OR ""Children""[Title/Abstract] OR ""Adolescents""[Title/Abstract] OR ""Adolescence""[Title/Abstract] OR ""teen*""[Title/Abstract] OR ""youth*""[Title/Abstract] OR ""paediatric*""[Title/Abstract] OR ""pediatric""[Title/Abstract] OR ""pediatry""[Title/Abstract]" " |
| #6 | """Adolescent""[Mesh]" Most Recent """Adolescent""[MeSH Terms]" " |
| #7 | """Pediatrics""[Mesh]" Most Recent """Pediatrics""[MeSH Terms]" " |
| #8 | "(((((((((((((Child[Title/Abstract]) OR (Adolescent[Title/Abstract])) OR (Pediatrics[Title/Abstract])) OR (Children[Title/Abstract])) OR (Adolescents[Title/Abstract])) OR (Adolescence[Title/Abstract])) OR (Teen*[Title/Abstract])) OR (Youth*[Title/Abstract])) OR (Paediatric*[Title/Abstract])) OR (pediatric[Title/Abstract])) OR (pediatry[Title/Abstract])) OR (""Child""[Mesh])) OR (""Adolescent""[Mesh])) OR (""Pediatrics""[Mesh])" """Child""[Title/Abstract] OR ""Adolescent""[Title/Abstract] OR ""Pediatrics""[Title/Abstract] OR ""Children""[Title/Abstract] OR ""Adolescents""[Title/Abstract] OR ""Adolescence""[Title/Abstract] OR ""teen*""[Title/Abstract] OR ""youth*""[Title/Abstract] OR ""paediatric*""[Title/Abstract] OR ""pediatric""[Title/Abstract] OR ""pediatry""[Title/Abstract] OR ""Child""[MeSH Terms] OR ""Adolescent""[MeSH Terms] OR ""Pediatrics""[MeSH Terms]" " |
| #9 | 9 "((""Dexmedetomidine""[Mesh]) OR ((((((((((((((((((((Dexmedetomidine[Title/Abstract]) OR (MPV-1440[Title/Abstract])) OR (MPV 1440[Title/Abstract])) OR (MPV1440[Title/Abstract])) OR (Precedex[Title/Abstract])) OR (bxcl 501[Title/Abstract])) OR (bxcl501[Title/Abstract])) OR (cepedex[Title/Abstract])) OR (da 9501[Title/Abstract])) OR (da9501[Title/Abstract])) OR (delos[Title/Abstract])) OR (dexamedetomidine[Title/Abstract])) OR (dexdomitor[Title/Abstract])) OR (dexdor[Title/Abstract])) OR (igalmi[Title/Abstract])) OR (primadex[Title/Abstract])) OR (sedadex[Title/Abstract])) OR (sileo[Title/Abstract])) OR (tpu 006[Title/Abstract])) OR (tpu006[Title/Abstract]))) AND ((((((((((((((Child[Title/Abstract]) OR (Adolescent[Title/Abstract])) OR (Pediatrics[Title/Abstract])) OR (Children[Title/Abstract])) OR (Adolescents[Title/Abstract])) OR (Adolescence[Title/Abstract])) OR (Teen*[Title/Abstract])) OR (Youth*[Title/Abstract])) OR (Paediatric*[Title/Abstract])) OR (pediatric[Title/Abstract])) OR (pediatry[Title/Abstract])) OR (""Child""[Mesh])) OR (""Adolescent""[Mesh])) OR (""Pediatrics""[Mesh]))" "(""Dexmedetomidine""[MeSH Terms] OR (""Dexmedetomidine""[Title/Abstract] OR ""MPV-1440""[Title/Abstract] OR ""MPV-1440""[Title/Abstract] OR ""MPV1440""[Title/Abstract] OR ""Precedex""[Title/Abstract] OR (""bxcl""[All Fields] AND ""501""[Title/Abstract]) OR ""bxcl501""[Title/Abstract] OR (""da""[All Fields] AND ""9501""[Title/Abstract]) OR ""delos""[Title/Abstract] OR ""dexamedetomidine""[Title/Abstract] OR ""dexdomitor""[Title/Abstract] OR ""dexdor""[Title/Abstract] OR ""igalmi""[Title/Abstract] OR ""sileo""[Title/Abstract] OR ""tpu 006""[Title/Abstract])) AND (""Child""[Title/Abstract] OR ""Adolescent""[Title/Abstract] OR ""Pediatrics""[Title/Abstract] OR ""Children""[Title/Abstract] OR ""Adolescents""[Title/Abstract] OR ""Adolescence""[Title/Abstract] OR ""teen*""[Title/Abstract] OR ""youth*""[Title/Abstract] OR ""paediatric*""[Title/Abstract] OR ""pediatric""[Title/Abstract] OR ""pediatry""[Title/Abstract] OR ""Child""[MeSH Terms] OR ""Adolescent""[MeSH Terms] OR ""Pediatrics""[MeSH Terms])" " |

**2.Cochrane**

| Search number | Query |
| --- | --- |
| #1 | MeSH descriptor: [Dexmedetomidine] explode all trees |
| #2 | (Dexmedetomidine): ti,ab,kw OR (MPV-1440):ti,ab,kw OR (MPV 1440):ti,ab,kw OR (MPV1440):ti,ab,kw OR (Precedex):ti,ab,kw |
| #3 | (bxcl 501): ti,ab,kw OR (bxcl501):ti,ab,kw OR (cepedex):ti,ab,kw OR (da 9501):ti,ab,kw OR (da9501):ti,ab,kw |
| #4 | (delos): ti,ab,kw OR (dexamedetomidine):ti,ab,kw OR (dexdomitor):ti,ab,kw OR (dexdor):ti,ab,kw OR (igalmi):ti,ab,kw |
| #5 | (primadex): ti,ab,kw OR (sedadex):ti,ab,kw OR (sileo):ti,ab,kw OR (tpu 006):ti,ab,kw OR (tpu006):ti,ab,kw |
| #6 | #1 OR #2 OR #3 OR #4 OR #5 |
| #7 | MeSH descriptor: [Child] explode all trees |
| #8 | MeSH descriptor: [Adolescent] explode all trees |
| #9 | MeSH descriptor: [Pediatrics] explode all trees |
| #10 | (Child): ti,ab,kw OR (Adolescent):ti,ab,kw OR (Pediatrics):ti,ab,kw OR (Children):ti,ab,kw OR (Adolescents):ti,ab,kw |
| #11 | (Adolescence): ti,ab,kw OR (Teen*):ti,ab,kw OR (Youth*):ti,ab,kw OR (Paediatric*):ti,ab,kw OR (pediatric):ti,ab,kw |
| #12 | (pediatry): ti,ab,kw |
| #13 | #7 OR #8 OR #9 OR #10 OR #11 OR #12 |
| #14 | #6 AND #13 |

**3.Embase**

| Search number | Query |
| --- | --- |
| #1 | 'dexmedetomidine'/exp |
| #2 | dexmedetomidine:ab,ti OR 'mpv 1440':ab,ti OR mpv1440:ab,ti OR 'mpv-1440':ab,ti OR precedex:ab,ti OR 'bxcl 501':ab,ti OR bxcl501:ab,ti OR cepedex:ab,ti OR 'da 9501':ab,ti OR da9501:ab,ti OR delos:ab,ti OR dexamedetomidine:ab,ti OR dexdomitor:ab,ti OR dexdor:ab,ti OR igalmi:ab,ti OR primadex:ab,ti OR sedadex:ab,ti OR sileo:ab,ti OR 'tpu 006':ab,ti OR tpu006:ab,ti |
| #3 | #1 OR #2 |
| #4 | 'child'/exp |
| #5 | 'adolescent'/exp |
| #6 | 'pediatrics'/exp |
| #7 | child:ab,ti OR adolescent:ab,ti OR pediatrics:ab,ti OR children:ab,ti OR adolescents:ab,ti OR adolescence:ab,ti OR teen*:ab,ti OR youth*:ab,ti OR paediatric*:ab,ti OR pediatric:ab,ti OR pediatry:ab,ti |
| #8 | #4 OR #5 OR #6 OR #7 |
| #9 | #3 AND #8 |

**4.Web of science**

| Search number | Query |
| --- | --- |
| #1 | 1: Dexmedetomidine (topic) OR MPV-1440 (topic) OR MPV 1440 (topic) OR MPV1440 (topic) OR Precedex (topic) OR bxcl 501 (topic) OR bxcl501 (topic) OR cepedex (topic) OR da 9501 (topic) OR da9501 (topic) OR delos (topic) OR dexamedetomidine (topic) OR dexdomitor (topic) OR dexdor (topic) OR igalmi (topic) OR primadex (topic) OR sedadex (topic) OR sileo (topic) OR tpu 006 (topic) OR tpu006 (topic) |
| #2 | Child (topic) OR Adolescent (topic) OR Pediatrics (topic) OR Children (topic) OR Adolescents (topic) OR Adolescence (topic) OR Teen* (topic) OR Youth* (topic) OR Paediatric* (topic) OR pediatric (topic) OR pediatry (topic) |
| #3 | #1 AND #2 |

**Table S2:** League table for the incidence of emergence agitation, with data presented as RR (95%CI).

| 0.3μg/kgDEX |  |  |  |  |  |
| --- | --- | --- | --- | --- | --- |
| 1.350 (0.023, 89.630) | 0.5μg/kgDEX |  |  |  |  |
| 3.303 (0.069, 185.60) | 2.419 (0.619, 10.67) | 1μg/kgDEX |  |  |  |
| 3.623 (0.066, 232.5) | 2.678 (0.558, 13.56) | 1.1 (0.303, 3.816) | 1.5μg/kgDEX |  |  |
| 6.220 (0.126, 375) | **4.49 (1.124, 24.37)*** | 1.863 (0.771, 5.218) | 1.692 (0.456, 7.523) | 2μg/kgDEX |  |
| 1.003 (0.022, 53.29) | 0.737 (0.191, 3.072) | **0.305 (0.154, 0.579)*** | **0.276 (0.085, 0.905)*** | **0.163 (0.059, 0.373)*** | NS |

**Table S3:** League table for PAED scores, with data presented as MD (95%CI).

| 2μg/kgDEX |  |  |  |
| --- | --- | --- | --- |
| 1.37 (-1.32,4.06) | 1μg/kgDEX |  |  |
| -1.69 (-5.90,2.52) | -3.06 (-7.32,1.21) | 0.3μg/kgDEX |  |
| -1.83 (-3.92,0.26) | **-3.20 (-5.39,-1.00)*** | -0.14 (-3.80,3.52) | NS |

**Table S4:** League Table for PACU stay time, with data presented as SMD (95%CI)

| 0.5μg/kgDEX |  |  |  |  |
| --- | --- | --- | --- | --- |
| -0.39 (-1.07, 0.28) | 1μg/kgDEX |  |  |  |
| **-1.03 (-1.78, -0.28)*** | -0.64 (-1.32, 0.03) | 1.5μg/kgDEX |  |  |
| **-0.79 (-1.46, -0.12)*** | -0.4 (-0.88, 0.09) | 0.25 (-0.43, 0.91) | 2μg/kgDEX |  |
| -0.2 (-0.86, 0.46) | 0.19 (-0.27, 0.65) | **0.83 (0.17, 1.5)*** | **0.59 (0.15, 1.02)*** | NS |

**Table S5:** League table for length of hospital stay, with data presented as SMD (95%CI).

| 0.5μg/kgDEX |  |  |  |  |
| --- | --- | --- | --- | --- |
| -3.87 (-16.37, 8.89) | 1μg/kgDEX |  |  |  |
| -6.64 (-19.12, 6.74) | -2.94 (-11.68, 7.85) | 1.5μg/kgDEX |  |  |
| -10.81 (-24.48, 1.16) | -6.36 (-19.13, 1.13) | -3.28 (-17.46, 2.97) | 2μg/kgDEX |  |
| -8.31 (-20.34, 3.49) | -4.32 (-12.72, 2.87) | -1.09 (-10.74, 4.51) | 1.79 (-3.17, 12.07) | NS |

**Table S6:** League table for postoperative gastrointestinal adverse reactions, with data presented as RR (95%CI).

| 1μg/kgDEX |  |  |  |
| --- | --- | --- | --- |
| 1.171 (0.074, 20.44) | 1.5μg/kgDEX |  |  |
| 1.026 (0.142, 5.93) | 0.888 (0.041, 12.48) | 2μg/kgDEX |  |
| 0.404 (0.097, 1.607) | 0.344 (0.028, 3.715) | 0.393 (0.096, 1.932) | NS |

**Table S7:** League table for parent's satisfaction, with data presented as SMD (95%CI).

| 1μg/kgDEX |  |  |
| --- | --- | --- |
| -0.75 (-1.77, 0.3) | 2μg/kgDEX |  |
| **1.46 (0.57, 2.36)*** | **2.21 (1.3, 3.1)*** | NS |

**Table S8:** League table for analgesics, with data presented as RR (95%CI)

| 0.5μg/kgDEX |  |  |  |  |
| --- | --- | --- | --- | --- |
| 1.293 (0.179, 8.954) | 1μg/kgDEX |  |  |  |
| 2.229 (0.197, 32.64) | 1.717 (0.207, 20.19) | 1.5μg/kgDEX |  |  |
| 1.188 (0.177, 10.24) | 0.918 (0.27, 4.141) | 0.543 (0.048, 5.394) | 2μg/kgDEX |  |
| 0.283 (0.042, 1.791) | **0.22 (0.082, 0.569)*** | **0.128 (0.011, 0.978)*** | **0.241 (0.061, 0.656)*** | NS |

**Table S9:** League table for postoperative pain scores, with data presented as SMD (95%CI)

| 1μg/kgDEX |  |  |
| --- | --- | --- |
| 1.47 (-8.58, 11.56) | 2μg/kgDEX |  |
| -4.33 (-13.08, 4.5) | -5.8 (-14.55, 2.91) | NS |

**Table S10:** SUCRA value of different doses of DEX

| **Outcomes** | **0.3μg/kg**  **DEX** | **0.5μg/kg**  **DEX** | **1μg/kg**  **DEX** | **1.5μg/kg**  **DEX** | **2μg/kg**  **DEX** | **NS** |
| --- | --- | --- | --- | --- | --- | --- |
| Emergence agitation | 31.80% | 28.95% | 63.52% | 68.35% | 90.91%^a^ | 16.47% |
| PAEDs | 30.34% | NR | 88.18%^a^ | NR% | 61.99% | 19.49% |
| PACU stay time | NR | 90.01% ^a^ | 55.89% | 6.66% | 21.19% | 76.26% |
| Postoperative length of hospital stay | NR | 86.62% ^a^ | 72.00% | 47.67% | 12.41% | 31.32% |
| Postoperative gastrointestinal adverse reactions | NR | NR | 61.77% | 64.31% ^a^ | 62.22% | 11.75% |
| Parental satisfaction | NR | NR | 52.19% | NR | 97.38% ^a^ | 0.43% ^c^ |
| Analgesics | NR | 49.60% | 61.64% ^a^ | 78.64% | 57.40% | 2.71% |
| Postoperative pain scores | NR | NR | 61.51% | NR | 77.27% ^a^ | 11.22% |

^a^ indicating the highest ranking

PAEDs, Pediatric Anesthesia Emergence Delirium score; PACU, Postanesthesia care unit; NR, not reported.


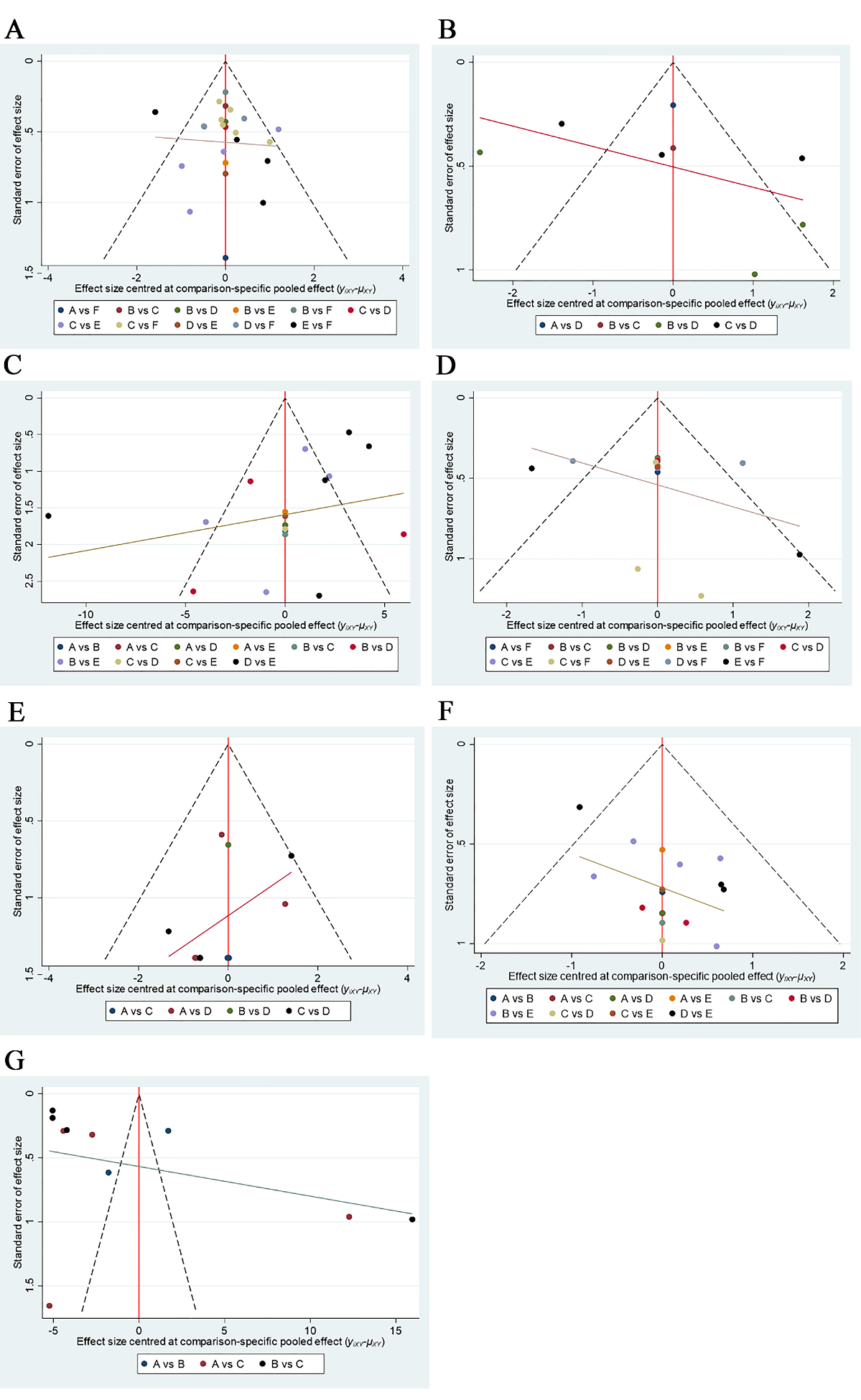


**Figure S1:** Funnel plots. Funnel plots for emergence agitation (A), PAED score (B), PACU stay time (C), extubation time (D), postoperative gastrointestinal adverse reactions (E), use of analgesics (F), and postoperative pain score (G).
